# Individual response in body mass and basal metabolism to the risks of predation and starvation in passerines

**DOI:** 10.1242/jeb.244744

**Published:** 2023-01-16

**Authors:** Juli Broggi, Jan-Åke Nilsson

**Affiliations:** ^1^ Department of Biology, Section of Evolutionary Ecology, University of Lund, S-223 62 Lund, Sweden; ^2^ Estación Biológica de Doñana (CSIC), Av. Américo Vespucio 26, 41092 Sevilla, Spain; ^3^ Departamento de Ecología Evolutiva, Museo Nacional de Ciencias Naturales - CSIC, C/José Gutiérrez Abascal 2, Madrid 28006, Spain

**Keywords:** Energy management, Optimal body mass theory, Food restriction, *Parus major*, Predation risk, Winter ecology

## Abstract

Wintering energy management in small passerines has focused on the adaptive regulation of the daily acquisition of energy reserves within a starvation–predation trade-off framework. However, the possibility that the energetic cost of living, i.e. basal metabolic rate (BMR), is being modulated as part of the management energy strategy has been largely neglected. Here, we addressed this possibility by experimentally exposing captive great tits (*Parus major*) during winter to two consecutive treatments of increased starvation and predation risk for each individual bird. Body mass and BMR were measured prior to and after each week-long treatment. We predicted that birds should be lighter but with a higher metabolic capacity (higher BMR) as a response to increased predation risk, and that birds should increase internal reserves while reducing their cost of living (lower BMR) when exposed to increased starvation risk. Wintering great tits kept a constant body mass independently of a week-long predation or starvation treatment. However, great tits reduced the cost of living (lower BMR) when exposed to the starvation treatment, while BMR remained unaffected by the predation treatment. Energy management in wintering small birds partly relies on BMR regulation, which challenges the current theoretical framework based on body mass regulation.

## Teaser

**Summary:** Wintering small birds faced with the risk of predation and starvation primarily respond by modulating energy reserves. However, wild great tits can also adjust their energy expenditure adaptively.

## INTRODUCTION

Wintering small passerines often face high energetic challenges to maintain body temperature within a living range. In particular, winter nights require an elevated energy expenditure for thermogenesis, with a concomitant increase in the internal reserves needed to fuel it (Broggi et al., 2019). Current understanding of winter energy management in small passerines focuses on the adaptive regulation of internal fat reserves as the main strategy to cope with changing environmental and ecological conditions (Brodin, 2007). The ‘optimal body mass’ (OBM) theory states that birds manage internal reserves on a short-term basis within a predation–starvation risk trade-off framework (Lima, 1986). Furthermore, the OBM implicitly assumes that most changes in body mass result from adaptive regulation of internal fat reserves (McNamara et al., 2005; Higginson, et al., 2012). According to the OBM theory, small birds should reduce their body mass to maximize their escape performance from predators, i.e. fit-for-flight hypothesis, but increase their reserve level to minimize their risk of starvation in the face of future increasing energy demands or decreasing food availability (Lima, 1986; Witter and Cuthill, 1993). As birds balance predation and starvation risks, they exhibit a clear pattern of daily mass accumulation, superimposed on a seasonal cycle known as winter fattening (Rogers and Rogers, 1990; but see Broggi et al., 2003, 2019). At the same time, winter-acclimatized birds exhibit a seasonal increase in their thermogenic capacity that is paralleled by an increase in the overall energy cost of living and basal metabolic rate (BMR) (Swanson, 2010; but see Petit et al., 2013). BMR is a measure of the physiological maintenance costs of a resting individual, which may change flexibly as the size/proportion of organs and tissues changes (Daan et al., 1990) in response to ecological and physiological circumstances (Kersten and Piersma, 1987; Broggi et al., 2007; McKechnie, 2008; Piersma and Van Gils, 2011). BMR is suggested to reflect individual energetic capacity (Nilsson, 2002; Sadowska et al., 2015) and behaviours related to resource acquisition and predation avoidance (Møller, 2009; Biro and Stamps, 2010; Mathot and Dall, 2013; Mathot and Dingemanse, 2015), albeit these links are necessarily considered indirect given the definition of BMR (Swanson et al., 2017). However, while energy management strategies have mostly focused on the acquisition and storage of fat reserves, changes in BMR are viewed as a necessary by-product of the adaptive modulation of other correlated traits, e.g. maximal metabolic rate, without the possibility of strategic adjustment to changing conditions, with few theoretical (Welton et al., 2002; Houston, 2010; Swanson et al., 2017) and empirical exceptions (Vézina et al., 2007, 2017; Broggi et al., 2019; Norin and Metcalfe, 2019). But, accumulating evidence suggests that BMR may respond differently to environmental conditions than other correlated metabolic traits (Dubois et al., 2016; Petit et al., 2013) and might play a direct role in energy management strategies (Halsey, 2018; Broggi et al., 2019).

On the one hand, according to the OBM theory, birds under high diurnal predation risk should be lean to take off and manoeuvre optimally, and because being heavier is associated with a longer exposure to predators while gathering food resources (MacLeod et al., 2005; but see Lind et al., 2010). On the other hand, OBM theory predicts that under reduced food predictability, birds should increase internal reserves (i.e. body mass) to be prepared for periods of food deprivation (Lima, 1986; Witter and Cuthill, 1993). However, if birds were to optimize their BMR, it could be predicted that under predation risk they should have a high capacity for metabolic output to maximally react to predator threats or to maximize the rate of food intake (i.e. an increase in BMR) (Mathot et al., 2016). Alternatively, birds in a starvation context could be predicted to reduce the cost of living to save energy (i.e. a decrease in BMR) (Wiersma and Verhulst, 2005; Swanson et al., 2017).

Body mass and BMR are phenotypically integrated traits (Pigliucci, 2003) with a substantial positive covariation (Broggi et al., 2009, 2019). Body mass adjustments represent an almost immediate response (hours) to present or anticipated conditions (Koivula et al., 2002). BMR, in contrast, requires a longer time to readjust that can take from several hours to a few days (Piersma and Lindström, 1997; Dubois et al., 2016). The opposite predictions for an optimal level of these traits under a predation–starvation context reveal an interesting scenario as the adjustment time may play a crucial role.

Here, we studied the body mass and BMR response of captive great tits (*Parus major*) to two consecutive treatments designed to independently increase perceived predation and starvation risk. Our aim was to evaluate the predictions of the OBM theory concerning responses in body mass, and how a potential adaptive adjustment of BMR to the experimental treatments affects the OBM response.

## MATERIALS AND METHODS

### Study species, trapping and housing

The great tit, *Parus major* Linnaeus 1758, is a small passerine (∼15–20 g) which is widespread throughout Eurasia. The species is a permanent resident of temperate deciduous forests around the Lund area (Sweden; 55°40'N, 13°25'E) and readily uses supplementary food during winter.

Great tits were captured at a permanent winter feeder by mist nets between December 2016 and early March 2017 and housed in individual cages for a 3 week period, before being released at the point of capture. Although great tits spend the winter in loose aggregations, flock members exhibit dominance hierarchies that lead to agonistic interactions in high densities, particularly in captive conditions (Gosler, 1993). The constant monitoring of the permanent feeder allowed most of the experimental birds to be recorded for a long period after the experiments concluded, which supported the safety of our experimental procedures despite the inevitable stress derived from any confinement procedures (Clinchy et al., 2004). The experiment was conducted on four batches of birds with 14 birds in each batch. The 14 birds composing a batch were mist-netted in a single day at the permanent feeder. Upon capture, all birds were ringed, tarsus length was measured, and their sex and age were determined (as yearling or older) following Jenni and Winkler (1994), before they were released into the outdoor individual cages. Two extra birds were captured and maintained in captivity in addition to the 14 birds within a batch for replacement in case there were any problems with acclimatization or escapes (two occasions). The outdoor aviary, just a few metres from the capture point, consisted of 16 individual cages organized in two rows of eight with a corridor in between that allowed access to each cage independently. Cages had a surface area of 2 m by 1.5 m and were 2 m high, and comprised a wooden structure with metallic mesh-net walls, with a wooden roof covering the central corridor and half the ground surface of the cages. A few cages were slightly bigger, but in either case the cages ensured full flight and movement capacity for birds. All cages were provided with two nestboxes for roosting and fresh tree branches for perching. A white fabric blind was attached to the mesh-net walls to prevent birds from seeing the neighbouring cages or the corridor. Food consisted of mixed unhusked peanuts, sunflower seeds and commercial fat balls provided *ad libitum*, except when birds were exposed to the starvation treatment (see below). Birds were habituated to these food types as they were the same as those provided at the permanent feeder where captures occurred. Mealworms (5 g) were added to the seed mixture. Food and water were replaced daily through a window that gave access to a small tray attached to the corridor wall of each cage, allowing replacement without entering the cage, and so minimizing disturbance. Birds were trapped within the aviary on three occasions during the experiment for night-time metabolic and body mass measurements (see below). Trapping was performed after dusk, by entering the aviary and manually capturing the roosting birds with the help of a red-light torch. Birds were released before dawn in the same roosting nestbox from which they were captured.

### Experimental design and treatments

After capture, birds were left 2–5 days (depending on the batch) for acclimation to housing conditions, and then measured (see below) for reference BMR and body mass (hereafter pre-treatment) before being exposed to two consecutive experimental treatments lasting 5–6 days each. The same measurements were taken after each treatment, exposing each bird to a series of three measurements, a procedure that lasted 14 days in total. All birds from each batch experienced the same order of experimental treatments, but the order was alternated between the batches. The fact that each individual experienced all treatments allowed each one to be its own control. This included controlling for individual variation in reaction to captivity that might influence their physiology and/or behaviour (Jacobs and McKechnie, 2014).

The two experimental treatments experienced by each bird aimed at increasing the risks of predation and starvation, by manipulating perceived predation risk and food predictability, respectively. The perceived risk of predation treatment (hereafter predation risk treatment) consisted of a daily combination of 2–3 different procedures from a total of 5 threatening experiences: (1) showing a cardboard model of a flying merlin (*Falco columbarius*) (Birdmobile^©^ Malcom Topp Patent) hanging from a nylon thread attached to a fishing line that was moved over their roof; (2) showing a stuffed stoat (*Mustela erminea*) and stuffed perched sparrowhawk (*Accipiter nisus*) on top of the cage roof; (3) chasing each bird inside the cage by one person; (4) scaring the bird by beating the walls from outside the cage (excluding the corridor walls); and (5) play-back of recorded alarm calls from different passerine species, and owl and hawk calls. All individuals were exposed to the same daily combination of procedures consecutively, so in addition to the time they were exposed to the treatment, they could also hear (but not see) their captive congeners alarming while experiencing the same procedure before and after themselves, until the end of the treatment, which lasted between 30 and 300 min altogether. Food predictability treatment (hereafter starvation risk treatment) consisted of a reduction to half (and a quarter on the last 2 days) of the total amount of food delivered daily to each bird. In addition to a reduction in the amount of food, mealworms were only added to the seed mix on alternate days. During the treatment, a feeding interruption was induced by temporarily removing the food tray between 30 and 240 min, at different times of the day. In all treatments, the combination of procedures in the predation risk treatment, food removal timing in the starvation risk treatment, and the start and duration of the entire treatments were randomized to increase unpredictability and avoid habituation.

### Individual measurements

A total of 56 birds (31 juveniles and 25 adults, 30 females and 26 males) were measured for body mass change in the four batches. Metabolic measurements were obtained from only three of the four batches comprising 42 individuals (26 juveniles and 16 adults, 22 females and 20 males) in groups of 7 individuals per night during the last 2 days of each treatment. After night-time captures in the aviaries, birds were transported in cloth bags by car to the laboratory facilities at Lund University (10 min drive), where birds were weighed to the nearest 0.1 g (Pesola spring balance) before entering the respirometer chambers for night-time metabolic measurements. Before dawn, birds were returned following the same procedure.

BMR is defined as the average minimal oxygen consumption under postabsorptive digestive conditions during the resting phase of the daily cycle of non-growing, non-reproductive animals at thermoneutrality (McNab, 1997). BMR measurements were performed in an open-circuit respirometer by measuring the oxygen consumption of each bird. Birds were placed individually inside air-sealed chambers (0.6 l) in a dark climate cabinet at a constant temperature of 25°C, well within the species' thermoneutral zone. The respirometer consisted of eight parallel identical channels in which pressurized outdoor air was directed to each chamber through mass-flow controllers (FlowBar8 Multichannel Mass Flow Meter, Sable Systems International, Las Vegas, NV, USA) at 300 ml min^−1^. Only 7 birds could be measured throughout the night as a baseline channel is needed for reference air. The water-scrubbed outgoing air from each individual chamber was sequentially redirected through an RM8 Multiplexer (Sable Systems) in cycles of 10 min to the CO_2_ (CA-10A, Sable Systems) and oxygen analysers (FC-10A, Sable Systems). Data were recorded by means of the UI2 (Sable Systems) interface and the supplier's software. Average minimum 5 min oxygen consumption was used to calculate BMR, following Hill (1972).

### Ethics

All procedures were conducted in agreement with the guidelines of the local ethical committee (permit: M 134-16).

### Statistical analyses

We analysed variation in body mass and BMR in relation to experimental treatment with generalized linear mixed models. In both sets of models, treatment was included as a fixed effect, with batch as a random factor and individual as repeated subject as implemented in proc GLIMMIX SAS 9.4 (SAS Institute Inc. 2009). Treatment included three levels: pre-treatment, predation and starvation. We tested for a potential bias arising from cage differences but as this variable was non-significant (*P*>0.5), we excluded it from further analyses. Furthermore, we analysed the effect of sex, age, tarsus length and treatment order, together with their interaction with treatment as explanatory variables. Additionally, a baseline value was also included as a covariate, by incorporating the pre-treatment BMR or body mass, respectively. Baseline body mass in pre-treatment birds was included as the body mass at capture. Models were estimated by REML and d.f. by the Satterthwaite method. Full models were reduced by sequential backward elimination of the least significant factor, starting with the interactions, until only significant factors (*P*<0.05) remained in the model. Differences between experimental categories were tested with *post hoc F*-tests on least square means from the implemented models. All variables are presented in tables together with the AICc value from the implemented model, and the *F*-values, d.f. and *P*-values corresponding to the least significant predictor to be removed. Parameter estimates ±s.e. are provided for continuous predictors. All *P*-values are two-tailed. All continuous variables fulfilled the requirements of normality.

## RESULTS

**Fig. 1.**
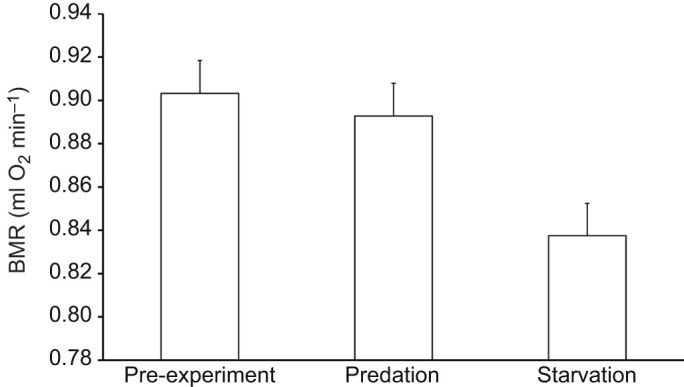
**Basal metabolic rate (BMR) of 42 captive great tits (*Parus major*) in response to predation and starvation treatment versus pre-treatment conditions.** Least square means from the final model are presented together with s.e.

In the final model, BMR was only significantly affected by the experimental treatment (*F*_2,79_=6.47, *P*=0.003; Fig. 1, Table 1). Birds maintained their BMR under predation risk with respect to pre-treatment values (estimate: 0.010±0.020, *t*_79_=0.53, *P*=0.60), whereas BMR after the starvation treatment decreased compared with both the pre-treatment (estimate: −0.066±0.020, *t*_79_=3.33, *P*=0.001) and predation treatment values (estimate: −0.055±0.020, *t*_79_=2.82, *P*=0.006) (Fig. 1). Age, sex and size parameters such as body mass and tarsus length had no significant influence (all *P*>0.1).

**Table 1.**
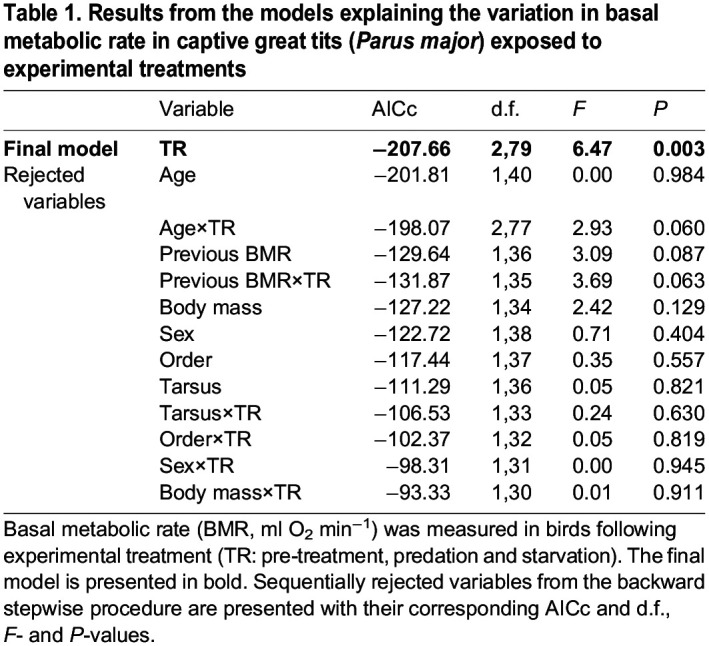
Results from the models explaining the variation in basal metabolic rate in captive great tits (*Parus major*) exposed to experimental treatments

**Fig. 2.**
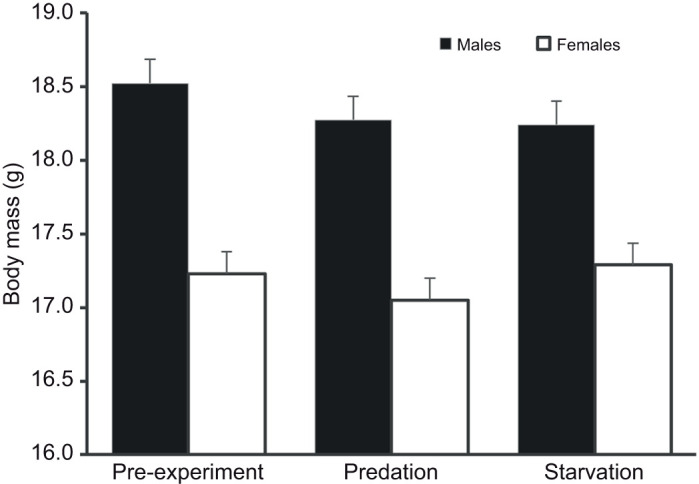
**Body mass for 56 female and male captive great tits (*P. major*) in response to predation and starvation treatment versus pre-treatment conditions.** Least square means from the final model are presented together with s.e.

**Fig. 3.**
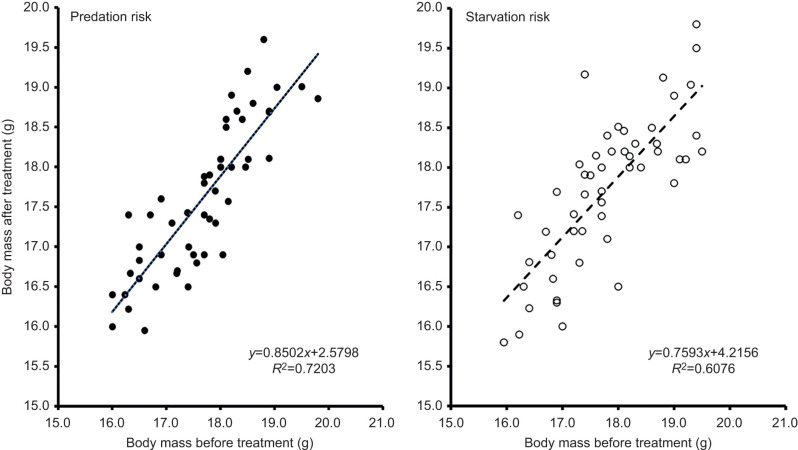
**Body mass after versus before predation and starvation treatment in 56 captive great tits (*P. major*).** Scatterplot of the raw values with corresponding tendency lines, regression equations and coefficients of determination. Predation risk values are plotted as filled circles and a solid tendency line, whereas starvation values are plotted as open circles with a dashed tendency line.

Body mass after the treatments was explained by sexual dimorphism and pre-treatment body mass (Table 2). Males were heavier than females (males: 17.9±0.088 g versus females: 17.4±0.073 g; *F*_1,49_=12.89, *P*<0.001; Fig. 2), and heavier birds at capture were also heavier after the treatments (slope: 0.659±0.067, *t*_69_=9.81, *P*<0.001; Fig. 3). Treatment had no significant effect on body mass (Table 2) and further *post hoc* testing indicated no significant change in body mass after the two treatments (estimate: −0.064±0.106, *t*_69_=−0.60, *P*=0.55).

**Table 2.**
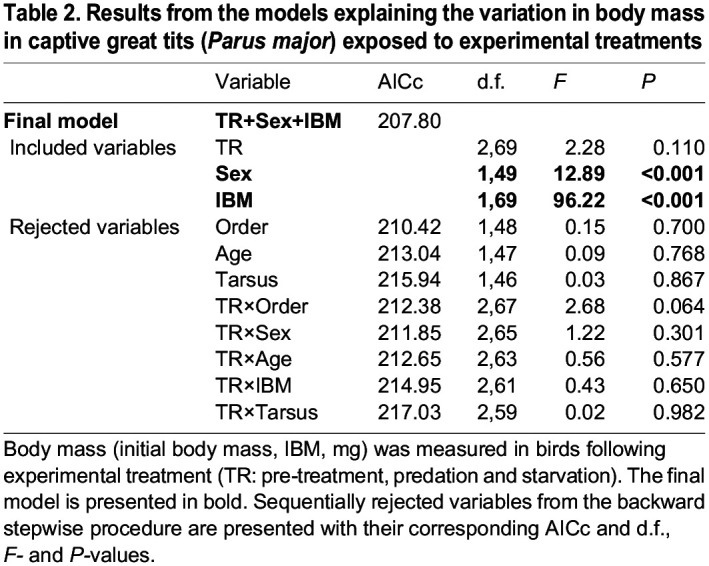
Results from the models explaining the variation in body mass in captive great tits (*Parus major*) exposed to experimental treatments

## DISCUSSION

Great tits exposed to starvation treatment responded by significantly changing BMR rather than body mass. Individuals reduced their BMR in a starvation as compared with a predation context and compared with pre-treatment values by ∼7% in just 5–6 days. Birds may benefit from a reduction of the overall costs of living when food predictability is impaired, which supports the hypothesis of BMR optimization. Body mass at dusk was dependent on sexual dimorphism, and mass at the start of the experiment, but otherwise it was not affected by any of the experimental treatments.

Food restriction experiments on birds, conducted over longer periods and within the thermoneutral zone, have also found a decrease in BMR, in line with our results (Mao et al., 2019; Zhang et al., 2018). However, these studies reported a paralleled decrease in BMR and body mass, suggesting the two traits are intrinsically related and thus vary in concert, rather than exhibiting independent strategic modulation. Great tits in our study responded to a reduced and more variable foraging success by reducing metabolic expenditure without changing body mass. These changes may result from organ size changes and/or tissue proportions as found by Piersma et al. (2004) in red knots (*Calidris canutus*) exposed to a change in diet, or metabolic intensity within organs (Rønning et al., 2008). Alternatively, birds may also have improved energy assimilation from digestion, as suggested by Bateson et al. (2021) in a food restriction experiment on European starlings (*Sturnus vulgaris*), in which birds were found to increase body mass although ingested food was reduced. Thus, temperate birds during winter may reduce the cost of living by decreasing BMR without sacrificing body reserves so they are prepared for sudden cold spells during a winter night.

Among the possible reasons for the non-significance of our predation risk treatment could be that we combined different kinds of perceived predation risks. As individual responses in both body mass and metabolism can be expected to differ according to the type of predator, even to the point that they show opposite patterns (Brodin, 2001), it is possible that optimal directional responses may have been mutually cancelled. The small-bird physiological response to different types of predators is a poorly known aspect of their energy management strategy and certainly deserves further study.

Previous research has shown body mass to respond to changes in perceived predation and starvation risk (Moiron et al., 2018), at least on a short-term basis (hours–days). However, given enough time (days–weeks), great tits may preferentially modulate their BMR, which suggests a much more strategic response than changing foraging trajectories of fat accumulation (Bonter et al., 2013). Body mass and BMR are phenotypically integrated as most tissues are metabolically active, and sum up in whole-organism BMR (Piersma, 2002). Further, as increases in energetic expenditure require an enlarged metabolic machinery and reserves to fuel it (Ksiazek et al., 2004; Rønning et al., 2007; Wone et al., 2009), changes in the two traits are almost inevitably positively correlated. However, the predicted values for body mass and BMR may not follow the same trajectory, which suggests a conflict between optimal levels of these two traits that may be dependent on the extent of environmental fluctuations and the time required for each trait to adjust. Evidence is accumulating that BMR is associated with behavioural differences among individuals (Houston, 2010; Norin and Metcalfe, 2019), and higher BMR levels can influence behaviours associated with resource acquisition, which in turn may increase exposure to predators (Biro and Stamps, 2010; Mathot et al., 2016). While these studies focus on inter-individual differences in the metabolic syndrome, our approach is intra-individual, showing that single individuals can make use of a regulation in BMR as an adaptive energy management strategy.

Even though the OBM theory traditionally has been at the core of the theoretical framework in energy management and foraging ecology in small birds (Brodin, 2007), the involvement of BMR should not be ignored in such regulation, albeit on different time scales. While body mass and the level of reserves may represent an almost immediate response to prevailing conditions (e.g. MacLeod et al., 2005), changes in BMR may reflect a longer-term strategic response to long-lasting and future conditions, as found in other taxa exposed to drastic changes in energetic demands and constraints over the annual cycle (Piersma, 2002). Thus, it is possible that, given enough time, regulation of BMR may facilitate an increased workload capacity when food is plentiful, or reduce overall metabolic costs under a closed energy budget (Deerenberg et al., 1998; Wiersma et al., 2005).

Here, we show that when confronted with a predation–starvation scenario, seasonally acclimatized small wintering passerines rely on the regulation of BMR. Thus, our results confirm that wintering birds at high latitudes manage not only their reserve levels but also their basal metabolism adaptively.
